# Camptothecin and Its Derivatives from Traditional Chinese Medicine in Combination with Anticancer Therapy Regimens: A Systematic Review and Meta-Analysis

**DOI:** 10.3390/cancers16223802

**Published:** 2024-11-12

**Authors:** Paul O. Odeniran, Paradise Madlala, Nompumelelo P. Mkhwanazi, Mahmoud E. S. Soliman

**Affiliations:** 1 Department of Veterinary Parasitology and Entomology, University of Ibadan, Ibadan 200001, Nigeria; drpaulekode@gmail.com; 2 HIV Pathogenesis Programme, School of Laboratory Medicine and Medical Science, The Doris Duke Medical Research Institute, University of KwaZulu-Natal, Durban 4000, South Africa; 3 Molecular Bio-Computation and Drug Design Lab, School of Health Sciences, University of Kwazulu-Natal, Durban 4000, South Africa

**Keywords:** anticancer drugs, camptothecin derivatives, objective response, meta-analysis, combinational therapy

## Abstract

**Background/Objectives**: Camptothecin (CPT) and its derivatives, irinotecan and topotecan, are integral components of cancer chemotherapy, often used in combination therapies. This meta-analysis evaluates the efficacy of CPT-based combination treatments in cancer patients. **Methods**: We systematically searched the literature database using the Preferred Reporting Items for Systematic Reviews and Meta-Analyses checklist for articles published between 2000 and 2022. Published studies were retrieved through an electronic search on the Web of Science, PubMed, and Google Scholar databases. A total of 138 studies were downloaded and examined, and 71 eligible studies were selected for meta-analysis after excluding studies that did not meet the inclusion criteria. **Results**: Ultimately, a total of 71 studies were included in the analysis, comprising non-small cell lung cancer (NSCLC), colorectal cancer (COLRC), oesophageal/gastric cancer (O/GC), and small cell lung cancer (SCLC). For NSCLC, the objective response rate (RR) was 31.8% (95% CI: 27.3–37.1%, *p* = 0.025), with irinotecan plus cisplatin showing significantly higher efficacy compared to other irinotecan-based combinations. In COLRC, irinotecan and 5-fluorouracil/leucovorin plus bevacizumab demonstrated superior efficacy with a RR of 44% (95% CI: 34–58, *p* < 0.001) and minimal haematological toxicity. In O/GC, irinotecan-based combinations showed an average RR of 43% (95% CI: 27–70, *p* < 0.001) and average overall survival (OS) and progression-free survival (PFS) rates of 10.2 and 5.5 months, respectively. For SCLC, irinotecan-based combinations had a higher control response than topotecan-based ones, while the latter exhibited higher rates of stable and progressive disease. The overall RR for SCLC was 45% (95% CI: 34.3–60.2, *p* < 0.001). **Conclusions**: The existing evidence underscored the potential of CPT-based combination therapy in various cancers. Among the various combinations discussed in this analysis, irinotecan plus cisplatin demonstrated the highest objective RR in 12 trials focused on NSCLC. This study provides valuable insights into potential treatment strategies for various types of cancer, emphasising the importance of personalised and tailored approaches to maximise efficacy and minimise adverse effects.

## Simple Summary

This study aims to systematically assess the efficacy and safety of camptothecin derivative-based drug combinations, such as irinotecan and topotecan, in treating various cancers, including non-small cell lung cancer (NSCLC), colorectal cancer, oesophageal/gastric cancer, and small cell lung cancer. By reviewing phase II and III clinical trials, the research focused on comparing objective response rates (RR), survival outcomes, and toxicity profiles across different chemotherapy regimens. Findings highlight that irinotecan combined with cisplatin showed superior RR and progression-free survival in NSCLC, while bevacizumab-irinotecan combinations demonstrated better outcomes in colorectal cancer. The results from this meta-analysis may inform clinical decision-making, guiding oncologists toward more effective treatment combinations for different cancer types and improving patient outcomes.

## 1. Introduction

Globally, cancer remains a significant health challenge, with nearly 10.0 million deaths and an estimated 19.3 million new cases reported in 2020 [1]. Female breast cancer ranks as the most diagnosed cancer, accounting for approximately 2.3 million new cases (11.7%), followed closely by lung (11.4%), colorectal (10.0%), prostate (7.3%), and stomach (5.6%) cancers. Despite advancements in treatment, lung cancer continues to be the leading cause of cancer-related deaths, claiming nearly 1.8 million lives (18%), followed by colorectal (9.4%), liver (8.3%), stomach (7.7%), and female breast (6.9%) cancers. Projections suggest a significant rise in cancer cases by 2040, with an anticipated 28.4 million new cases globally, representing a 47% increase from 2020 [1]. In 2020, lung cancer accounted for 2.21 million new cases and resulted in 1.8 million deaths globally. The age-standardised incidence rate stood at 22.4 per 100,000 individuals, with a higher incidence among males (31.5 per 100,000) than females (14.6 per 100,000). Similarly, the age-standardised mortality rates were 18.0 per 100,000 overall, with males experiencing a higher mortality rate (25.9 per 100,000) compared to females (11.2 per 100,000) [2]. This meta-analysis investigates the effective treatment combinations for different cancer types using camptothecin (CPT) and its derivatives from traditional Chinese medicine (TCM).

The utilisation of complementary and alternative medicines (CAMs) among cancer patients has increased in popularity. Notably, studies indicate that approximately one-third of individuals diagnosed with cancer incorporate CAMs into their treatment regimen [3]. Over the past five decades, extensive clinical research has highlighted the potential benefits of TCM in cancer therapy. Evidence suggests that TCM may reduce treatment-related toxicity, enhance the effectiveness of radiotherapy and chemotherapy, prolong survival in advanced cancer patients, and aid in preventing and managing recurrence and metastasis [4]. Given its widespread use, further research is imperative to elucidate TCM’s clinical efficacy and safety profile in cancer management.

Camptothecin, derived from *Camptotheca acuminata*, a tree native to China and a recognised traditional Chinese medicine, garnered interest in cancer treatment research in the late 1950s and mid to late 1960s [5]. Despite its potent anticancer properties, CPT’s unpredictable pharmacological behaviour and adverse effects (AEs) hampered its clinical success. Efforts to enhance CPT’s pharmacological profile and reduce side effects led to the development of semi-synthetic and synthetic analogues [6]. Topotecan and irinotecan, the first Food and Drug Administration-approved analogues, are now widely used in chemotherapy. Irinotecan is indicated for metastatic colorectal carcinoma, while topotecan serves as a second-line treatment for ovarian and small cell lung cancer [6]. The incorporation of front-line chemotherapy in cancer treatment strategies has expanded, particularly for advanced disease stages, through multimodal approaches [7]. Numerous clinical trials spanning five decades have explored CPT derivatives in cancer treatment. Previous studies have focused on the pharmacological profile, clinical evaluations, structural modifications, and therapeutic efficacy of CPT and its various forms [8,9,10]. However, no study has conducted a detailed, case-by-case clinical analysis of CPT analogues to thoroughly assess their combinational efficacy and identify the most suitable options for specific cancer therapies. This comprehensive meta-analysis aims to systematically assess the efficacy and safety of CPT derivative-based combinations in clinical trial settings, offering insights into their role in cancer therapy and the best choices at clinicians’ disposal.

## 2. Methods

### 2.1. Literature Searches

The study adhered to the Preferred Reporting Items for Systematic Reviews and Meta-Analyses criteria (PRISMA) [11] to ensure comprehensive inclusion of relevant data. A systematic literature search was conducted across multiple databases, including Web of Science, PubMed, and Google Scholar. Key search terms such as “camptothecin”, “irinotecan”, “topotecan”, “response rate”, “cancer”, “colorectal”, “gastric,” “oesophagus”, “small cell”, “non-small cell”, “lung cancer”, and “stomach” were employed.

Only studies published between January 2000 and December 2022 were considered, and inclusion criteria stipulated that articles must have full texts or abstracts available in English. Trials must be completed within the specified timeframe, objective response rates (RR) must be reported, clear details on the chemotherapeutic regimen must be provided, and a sample size of at least 10 must be used for statistical analysis. Articles lacking these characteristics were excluded from the study. The systematic review followed the recommendations of the PRISMA in Table S1 (Prisma 2020 checklist). The protocol has not been registered.

### 2.2. Quality of the Studies

The risk of bias within the included studies was assessed using a quality evaluation checklist based on criteria earlier outlined [12]. Each item was rated as yes, no, or uncertain and graded on a scale from 1 to 10. The checklist covered the following topics: clarity of study objectives, mention of therapy duration, documentation of gender distribution, mention of previous treatment, disclosure of sample size and RR, evaluation of progression-free and patient survival times, specification of substance class, division of examined subjects into subgroups, accurate description of therapy duration, and mention of patients’ performance status. These criteria were employed to systematically assess the quality and potential biases within the selected studies.

### 2.3. Data Extraction

Study characteristics (first author’s name, year of publication, impact factor, response rate, including control response (CR), partial response (PR), stable disease (SD), and progression disease (PD)); median age, prior therapies, toxicity effects, survival time, progression-free survival, performance status, and overall survival time (Supplementary File S1) were all noted.

### 2.4. Statistical Analysis

To evaluate the efficacy of cancer chemotherapy, the primary outcome measure utilised was the objective RR, presented alongside its corresponding 95% confidence interval (95% CI). All descriptive analyses employed 95% confidence intervals, while the raw data were processed in Microsoft Excel. In certain instances, standard deviation (StD) and standard error of the mean (SEM) were also examined. The objective response encompasses control and partial response, whereas the disease control rate incorporates control response, partial response, and stable disease. These metrics provide valuable insights into the effectiveness of cancer chemotherapeutic interventions. The response rate from each trial was combined using the Microsoft Excel meta-analysis program METAXL version 3.1 [13,14]. The quality effects model computed the estimated response rate and 95% confidence intervals (CI). According to this model’s quality ratings, research is ranked according to its quality and sample size [13]. Prevalence information was taken from various sub-group analyses. I2 (degree of inconsistency) was used to evaluate heterogeneity in the study (Cochran’s Q). According to Barendregt and Doi [14], the I2 values of 25%, 50%, and 75% indicated low, moderate, and high degrees of heterogeneity, respectively. Both Doi plots and funnel plots were used to evaluate the risk of bias. According to Barendregt and Doi [14], a Luis Frya-Kanamori (LFK) score greater than 2 denotes significant asymmetry (publication bias). Plotting the z-score versus double arcs in the prevalence of the analysed publications yields the LFK index. The total number of sampled population (*n* − 1), where “*n*” is the sample population, is denoted by the degree of freedom (df) in the text.

## 3. Results

### 3.1. Meta-Analysis Trial Flow

**Figure 1:**
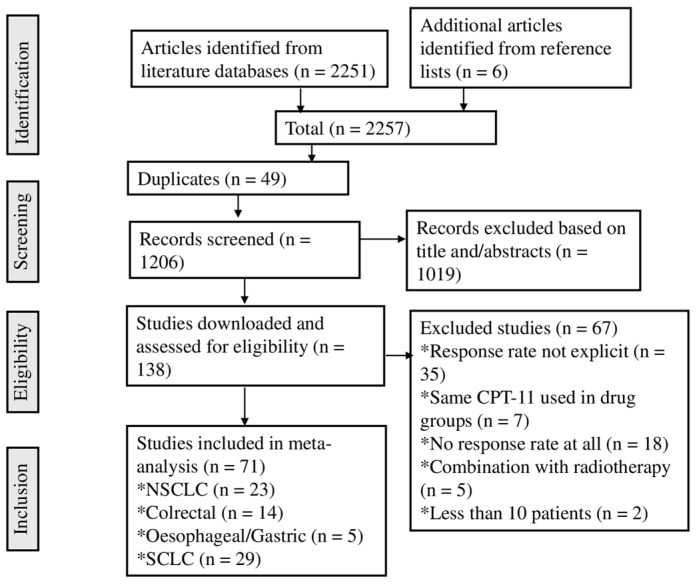
Flow diagram on the exclusion and inclusion of articles in a systematic approach for the camptothecin derivative-based combinations on some specific cancer treatments.

The literature database searches yielded 2257 articles, including references from relevant studies (Figure 1). After diligent observation, 1051 studies were removed as they were unrelated to the subject being considered. Thereafter, 1019 articles were excluded based on screening titles and abstracts from the initial 1206 records. Additionally, 49 duplicate studies were identified. Upon review of the full texts of the remaining articles, 67 did not meet the inclusion criteria out of the 138 downloaded. Ultimately, 71 studies were included in the analysis, comprising 23 for NSCLC, 14 for colorectal cancer (COLRC), 5 for oesophageal/gastric cancer (O/GC), and 29 for SCLC.

#### Non-Small Cell Lung Cancer (NSCLC) Analysis with Irinotecan-Based Combinations

**Figure 2:**
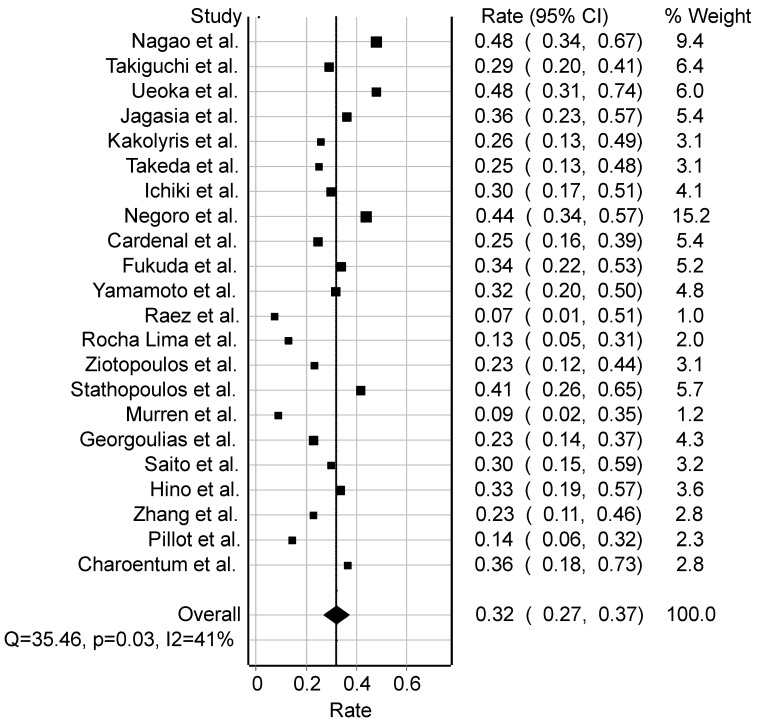
Forest plot of studies on NSCLC among 1092 patients (Georgoulias [7], Nagao [15], Takiguchi [16], Ueoka [17], Jagasia [18], Kakolyris [19], Takeda [20], Ichiki [21], Negoro [22], Cardenal [23], Fukuda [24], Yamamoto [25], Raez [26], Rocha Lima [27], Ziotopoulos [28], Stathopoulos [29], Murren [30], Saito [31], Hino [32], Zhang [33], Pillot [34], Charoentum [35]). Abbreviations: NSCLC, non-small cell lung cancer; Q, measure of heterogeneity; *p*, *p*-value; I2, degree of inconsistency; CI, confidence interval.

**Figure 3:**
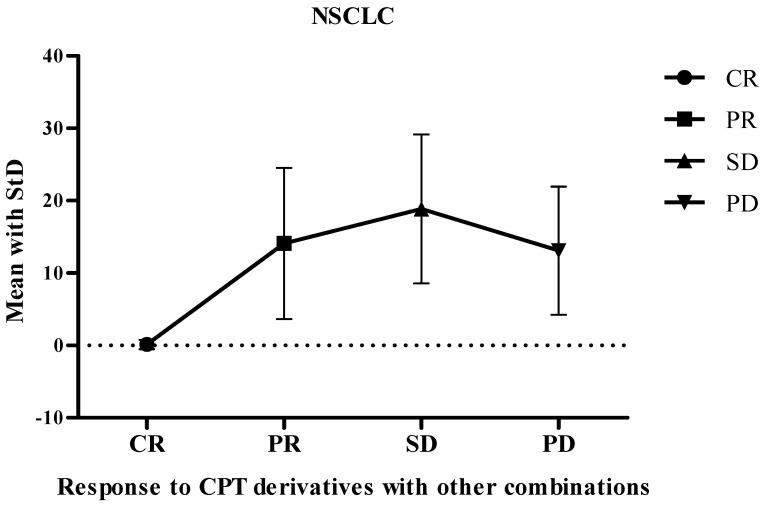
Responses to irinotecan combinations against NSCLC from patient metadata. Abbreviations: CPT, camptothecin; CR, control response; NSCLC, non-small cell lung cancer; PD, progressive disease; PR, partial response; SD, stable disease; StD, standard deviation.

The objective response rate in studies focusing on non-small cell lung cancer was determined to be 31.8% (*n* = 23 studies), based on 1092 individuals assessed for response (Figure 2). The overall pooled control response rates for CR, PR, SD, and PD were calculated as follows: CR = 0.15 (StD = 0.6708, SEM = 0.15), PR = 13.6 (StD = 10.84, SEM = 2.42), SD = 18.3 (StD = 10.70, SEM = 2.52), and PD = 13 (StD = 9.35, SEM = 2.20), respectively (Figure 3). The overall analysis revealed heterogeneity among the studies (RR = 31.8%, 95% CI: 27.3–37.1%, I2 = 40.8%, Cochran’s Q = 35.5, *p* = 0.025, df = 21, Q-index = 15.0).

### 3.2. NSCLC Study Analyses

The average median age across the studies examined was 62 years (*df* = 19). The one-year overall survival rate (OS), as observed in four studies, was determined to be 22.2% (17.1–28.1). Evaluating the overall statuses of the examined studies based on the Eastern Cooperative Oncology Group Performance Status (ECOG), the results were as follows: ECOG 0 = 30.0% (95% CI: 26.3–33.9, StD = 0.1276, SEM = 0.0191); ECOG 1 = 60.0% (95% CI: 55.9–64.1, StD = 0.1419, SEM = 0.020); and ECOG 2 = 12.2% (95% CI: 9.9–14.8, StD = 0.074, SEM = 0.0119). Patients with an ECOG status of IIIB (24.7%) showed significantly lower rates compared to those with IV status (74.5%) (χ^2^ = 507.5; *p* < 0.0001). Recurrent patients accounted for 0.8% of the total. The median survival time estimated across 20 studies was 10.5 months, while the average progression-free survival time, evaluable in 15 studies, was 4.9 months. Toxicity reports were limited to patients experiencing grade III and IV AEs. The observed AEs and their respective percentages (with 95% CI and sample sizes) were as follows: nausea (13.5%, 95% CI: 11.1–16.2, *n* = 98/727, *df* = 14), diarrhoea (15.6%, 95% CI: 13.2–18.3, *n* = 129/826, *df* = 16), neutropenia (39.3%, 95% CI: 35.9–42.8, *n* = 311/791, *df* = 17), anaemia (12.2%, 95% CI: 9.9–14.7, *n* = 92/755, *df* = 16), leukopenia (22.0%, 95% CI: 18.1–26.4, *n* = 91/413, *df* = 7), anorexia (25.0%, 95% CI: 16.4–35.4, *n* = 22/88, *df* = 1), thrombocytopenia (12.1%, 95% CI: 9.8–14.7, *n* = 87/718, *df* = 14), fatigue (8.1%, 95% CI: 5.4–11.4, *n* = 27/335, *df* = 6), and vomiting (9.9%, 95% CI: 5.4–16.4, *n* = 13/131, *df* = 2).

### 3.3. Irinotecan Drug Combinations and Responses

More studies were observed investigating the combination of irinotecan plus cisplatin against NSCLC compared to other drug combinations, as shown in Table 1. The average objective response rate was also higher in the irinotecan plus cisplatin combination compared to other combinations. The RR analysis indicated that irinotecan plus cisplatin had a significantly higher response rate compared to combinations such as irinotecan plus gemcitabine, irinotecan plus docetaxel, and irinotecan plus carboplatin, but not significantly different (*p* > 0.05) from others like irinotecan plus paclitaxel and irinotecan plus ifosfamide. The median age of patients during chemotherapy was statistically similar across all combinations and did not appear to affect the efficacy of the drugs. Survival analysis revealed that irinotecan plus gemcitabine and irinotecan plus paclitaxel had the lowest OS compared to other irinotecan combinations in the studies that reported higher OS, while irinotecan plus docetaxel exhibited the highest OS. Additionally, progression-free survival (PFS) was notably higher for irinotecan plus cisplatin and irinotecan plus carboplatin than other combinations.

### 3.4. Drug Toxicity Among Patients in Grades III and IV

For all irinotecan combinations used against NSCLC, neutropenia is the most severe drug toxicity observed in all the irinotecan-based combinations (Table 2). The low toxicity observed in irinotecan *plus* ifosfamide could be due to only one study examined in that group.

### 3.5. Colorectal Cancer

**Figure 4:**
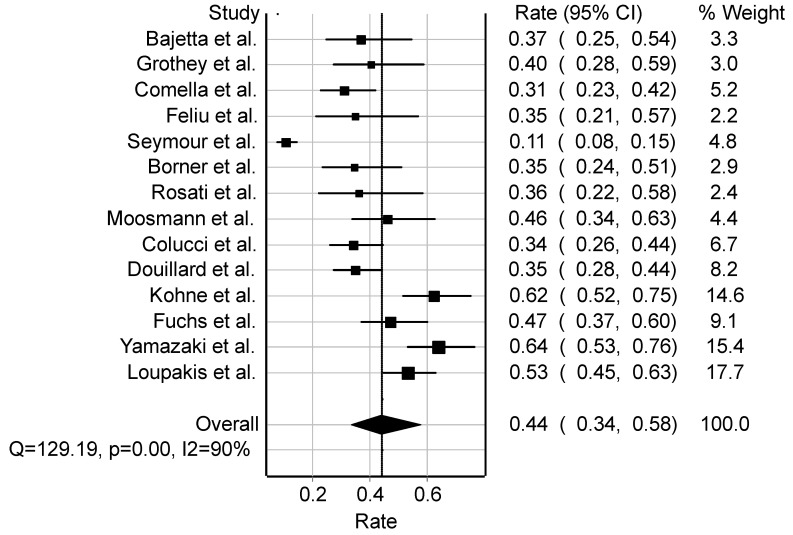
Forest plot of studies on COLRC among 2018 patients using camptothecin-based derivatives with other drugs (Bajetta [36], Grothey [37], Comella [38], Feliu [39], Seymour [40], Borner [41], Rosati [42], Moosmann [43], Colucci [44], Douillard [45], Kohne [46], Fuchs [47], Yamazaki [48], Loupakis [49]). Abbreviations: COLRC, colorectal cancer; Q, measure of heterogeneity; *p*, *p*-value; I2, degree of inconsistency; CI, confidence interval.

The objective response rate of camptothecin derivatives with or without other drugs against COLRC demonstrates a pooled rate of 44% (95%CI: 34–58) (Figure 4), encompassing 2018 patients. However, the analysis indicates significant heterogeneity between studies (Q = 129.2; *p* = 0.001; I2 = 90%). The breakdown of response categories reveals a CR of 4.0% (95%CI: 3.2–5.0), PR at 35.4% (95%CI: 33.3–37.6), SD at 30.6% (95%CI: 28.5–32.8), and PD at 20.5% (95%CI: 18.6–22.4). The median age of the examined studies is 63 years. The OS stands at 19.1 months, while PFS is estimated at 10.1 months across the studies. Notably, approximately 49.1% (95CI: 46.0–52.2) of patients exhibit metastases at one site, 34.6% (95%CI: 30.8–38.4) at two sites, and 18.4% (95%CI: 15.5–21.5) at more than two sites. Considering the performance status of patients, ECOG 0 is reported in 66.1% (95%CI: 63.4–68.7, *n* = 9 studies), ECOG 1 in 36.9% (95%CI: 33.9–40.0, *n* = 8 studies), and ECOG 2 in 4.5% (95%CI: 3.1–6.4, *n* = 6 studies).

### 3.6. Irinotecan-Based Combinations Response

Most studies focused on folinic acid (also known as leucovorin, calcium folinate, or FA), fluorouracil (also known as 5FU), and irinotecan (FOLFIRI) combinations for the treatment of COLRC. Notably, combining bevacizumab with irinotecan and 5-fluorouracil/leucovorin (FOLFIRI) demonstrated significantly higher OS, PFS, RR, and disease control rate (DCR) comparable safety profiles (Table 3). Among irinotecan-based regimens for first-line metastatic COLRC, an infusion schedule of fluorouracil (FU) should be preferred.

### 3.7. Drug Toxicity

The toxicity was only for grade III and IV patients, with severity signs of anaemia, neutropenia, and leucopenia, most pronounced with patients treated with BEVFOLFIRI. Diarrhoea and vomiting were most severe with CAPIRI (Table 4). In all the analyses, AEs between FOLFIRI and CAPIRI were comparable, as patients’ reactions were similar.

### 3.8. Oesophageal/Gastric Cancer

**Figure 5:**
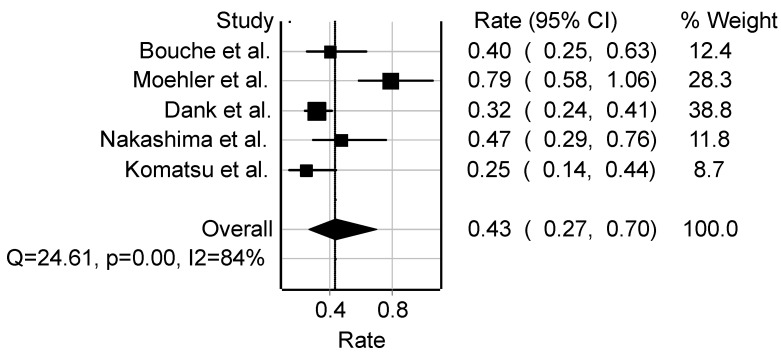
Forest plot of studies conducted on oesophageal/gastric cancer (Bouche [50], Moehler [51], Dank [52], Nakashima [53], Komatsu [54]). Abbreviations: Q, measure of heterogeneity; *p*, *p*-value; I2, degree of inconsistency; CI, confidence interval.

The pooled rate from six studies on the effect of irinotecan-based chemotherapy with other drug combinations revealed a response rate of 43% (95%CI: 27–70) among 355 patients (Figure 5). The most common regimen across studies is FOLFIRI (LV 200 mg/m^2^ i.v. followed by FU 400 mg/m^2^–600 mg/m^2^ i.v. on days 1 and 2 every 14 days plus irinotecan 180 mg/m^2^). The study showed heterogeneity (*Q* = 24.6, *p* < 0.0001, I2 = 84%) (Figure 5). Observed responses across studies include CR = 2.2%, PR = 36.2%, SD = 27.0%, and PD = 20.5%. The overall median age was 63.4 years in the five studies. A total of 16.8% of 137 patients have prior surgical resection before chemotherapy. Based on performance status, ECOG 0 = 70.6%, ECOG 1 = 27.2%, and ECOG 2 = 2.2%. The median survival time was 10.2 months, while progression-free survival time was 5.5 months. Based on metastatic sites, patients with one organ metastatic sites were 39.7% (95%CI: 33.8–45.8), and two organs were 39.0% (95%CI: 33.1–45.1). while greater than two organs were 21.3% (95%CI: 17.0–26.8). Effects of toxicity were reported in nausea 10.3% (95%CI: 7.4–14.0), diarrhoea 22.4% (95%CI: 18.1–27.0), stomatitis 4.7% (95%CI: 2.4–8.0), anaemia 12.1 (95%CI: 8.7–16.2), neutropenia 26.3% (95%CI: 21.8–31.1), leukopaenia 14.6% (95%CI: 10.1–20.0), anorexia 5.6% (95%CI: 3.2–9.0), and vomiting 7.8% (95%CI: 3.5–14.9).

### 3.9. Small Cell Lung Cancer (SCLC)

**Figure 6:**
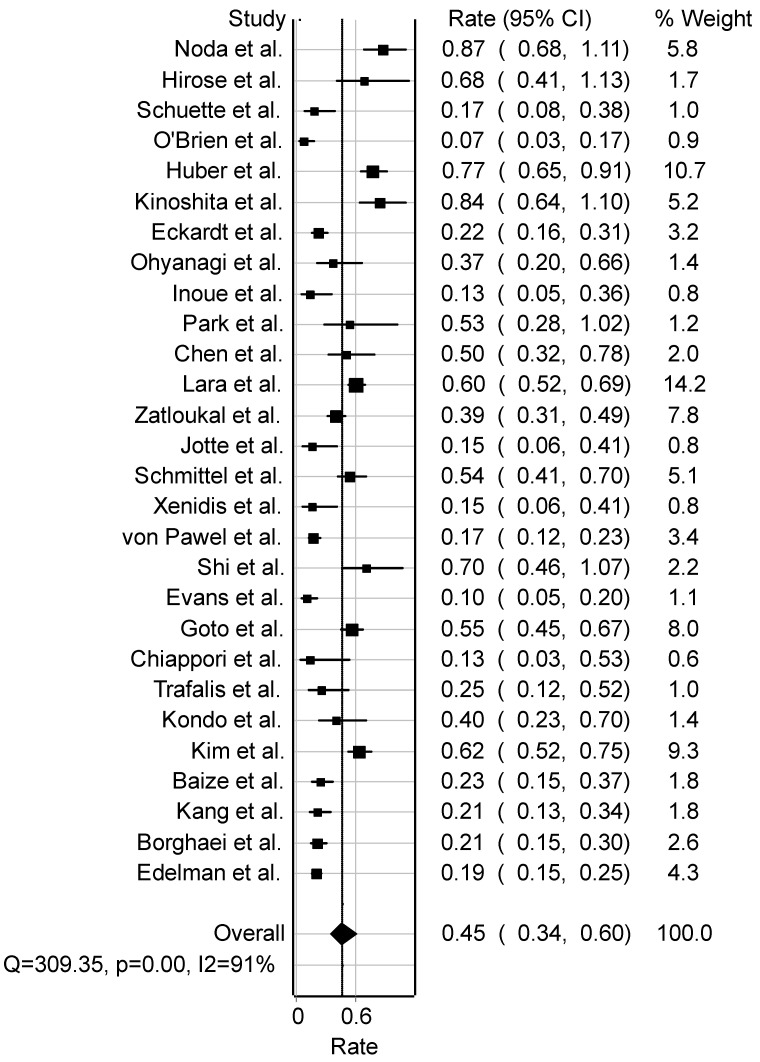
Forest plot of studies on SCLC patients (Noda [55], Hirose [56], Schuette [57], O’Brien [58], Huber [59], Kinoshita [60], Eckardt [61], Ohyanagi [62], Inoue [63], Park [64], Chen [65], Lara [66], Zatloukal [67], Jotte [68], Schmittel [69], Xenidis [70], von Pawel [71], Shi [72], Evans [73], Goto [74], Chiappori [75], Trafalis [76], Kondo [77], Kim [78], Baize [79], Kang [80], Borghaei [81], Edelman [82]). Abbreviations: SCLC, small cell lung cancer; Q, measure of heterogeneity; *p*, *p*-value; I2, degree of inconsistency; CI, confidence interval.

From 2002 to 2022, the chemotherapeutic treatment of small cell lung cancer (SCLC) predominantly involved two major camptothecin-based drugs: irinotecan and topotecan, either administered alone or in combination with other drugs. A forest plot analysis revealed a pooled overall response rate of 45.4% (95%CI: 34.3–60.2) across 28 studies involving 2704 patients (Figure 6). However, notable asymmetry and heterogeneity were observed among the studies (Q = 309.4, *p* < 0.001, I2 = 91%) (Figure 6). Specifically, the SCLC analysis indicated control response rates of 4.0% (95%CI: 3.3–4.9), partial response rates of 32.7% (95%CI: 30.8–34.8), stable disease rates of 24.3% (95%CI: 22.5–26.1), and progressive disease rates of 16.0% (95%CI: 15.0–17.6). The median age across all 29 studies encompassing 2704 patients was 64 years. Performance status assessed by the Eastern Cooperative Oncology Group (ECOG) revealed ECOG 0 at 27.2% (95%CI: 25.0–29.6), ECOG 1 at 64.3% (95%CI: 61.8–66.8), and ECOG 2 at 8.8% (95%CI: 7.4–10.4). The median survival time across all SCLC studies was 8.9 months, with a progression-free survival of 4.5 months. Notably, 46.4% (95%CI: 42.5–50.3) of patients had undergone prior radiotherapy, while surgical resection was reported in 14.9% (95%CI: 10.6–20.1) from two studies. The analysis of toxicity revealed varying incidences, including nausea (7.5%), diarrhoea (10.6%), anaemia (22.8%), neutropenia (51.0%), leukopenia (41.7%), anorexia (3.1%), thrombocytopenia (32.0%), fatigue (15.2%), and vomiting (5.1%).

### 3.10. Comparison of Topotecan-Based Combinations with Irinotecan-Based Combinations Against SCLC from 29 Studies Analysed

A total of 14 and 16 studies investigated the efficacy of topotecan and irinotecan-based combinations in chemotherapy for SCLC. The average response rate was significantly higher in studies utilising irinotecan than those employing topotecan. However, there was no significant difference in the DCR. The median age was comparable between the two groups. Overall survival rate and PFS were higher in irinotecan-based combinations. Drug response analysis revealed a higher control response in irinotecan-based combinations compared to topotecan, although the difference was not significant. Additionally, partial response was significantly higher in irinotecan-based combinations than topotecan (Table 5). Conversely, topotecan-based combinations exhibited a higher rate of stable disease and progressive disease, statistically significant compared to irinotecan-based combinations. Toxicity analysis indicated that irinotecan-based combinations had a greater impact on non-haematological parameters such as anaemia and diarrhoea, while topotecan-based combinations showed higher effects on haematological parameters.

### 3.11. Sensitivity Test on Analysis

For sensitivity tests of each MetaXL analysis, the stability and dependability of the processed articles were assessed. The Doi plot (LFK index) used for sensitivity analysis eliminated any chance of major bias in the study. NSCLC provides an LFK index of −3.17, colorectal cancer analysis shows −2.07, O/GC LFK index is −0.30, and SCLC analysis LFK index shows −4.95. All except O/GC analysis show major asymmetry.

## 4. Discussion

The meta-analysis focused on phase II and III studies investigating the efficacy of irinotecan and topotecan in combination with other drugs (such as cisplatin, carboplatin, fluorouracil, etc.) across four cancer types (SCLC, NSCLC, COLRC, and O/GC), with a total cohort of 6169 patients.

Irinotecan-based regimens, augmented with additional medications, predominated in the treatment of NSCLC, yielding an average RR of 32%. However, the CR rate was notably low, with less than 0.2% reported across 23 studies. Notably, irinotecan administration has been linked to suboptimal CR outcomes, particularly in patients classified as grade IV [26,30,34]. The analysis underscored the significant incidence of severe AEs, encompassing haematologic and non-haematologic manifestations commonly associated with CPT chemotherapies [82,83]. Irinotecan plus cisplatin had the best OS and PFS time, which could be why more studies were conducted on this combination for NSCLC. The analysis revealed that irinotecan plus cisplatin demonstrated the highest objective RR among the combinations studied in 12 trials focused on NSCLC (Table 1). While irinotecan plus ifosfamide exhibited the highest DCR%, its relatively low RR% might deter its widespread use. Notably, the chemotherapy regimen comprising irinotecan plus cisplatin consistently outperformed other combinational regimens across multiple parameters, including objective response rate, median time to tumour progression, median survival time, and 1-year survival in the studies analysed for NSCLC. This suggests that the irinotecan plus cisplatin combination may be the preferred treatment option among the examined regimens.

In studies involving metastatic COLRC patients, phase III randomised trials indicated improved PFS with CPT derivative-based combination therapies. Notably, CAPIRI and FOLFIRI emerged as the most frequently utilised drugs, according to literature databases. Compared to other combination therapies, BEVFOLFIRI exhibited superior OS and PFS. However, inconsistencies in OS and PFS observed in studies focusing on FOLFIRI may be attributed to data from phase II trials [47]. Overall, no significant difference was observed in the RR and DCR between FOLFIRI and CAPIRI. The enhanced outcomes associated with BEVFOLFIRI may be attributed to including Bevacizumab, a monoclonal antibody known for inhibiting the formation of blood vessels crucial for cancer growth. Additionally, the higher dose of irinotecan (150–180 mg/m^2^) in BEVFOLFIRI compared to other regimens may contribute to its efficacy. Notably, severe AEs such as anaemia and neutropenia were more pronounced with BEVFOLFIRI, while CAPIRI was associated with more severe diarrhoea compared to previous medication options. The high proportion of patients with an ECOG performance status of zero (66.1%) suggests that a more intensive treatment regimen may be feasible without compromising patient selection criteria. Overall survival notably improved, underscoring the importance of incorporating irinotecan into the treatment regimen. Esteemed guidelines and established data advocate for a maintenance phase and continuum of care for individuals grappling with metastatic colorectal cancer [84,85,86].

The reviewed trials demonstrated objective response rates (RR) falling within the reported range (25–79%) for phase II and III investigations in O/GC. While one study encompassed both stomach and oesophageal cancer, four studies focused solely on gastric cancer. These irinotecan-based combinations show promise, as evidenced by the average OS and PFS rates of 10.2 and 5.5 months, respectively. A detailed examination reveals a low CR rate with a moderately robust partial response, consistent with prior research findings [87,88,89]. With oncologists prioritising the extension of meaningful survival, consideration of treatment-related toxicity is imperative. Patients with shorter survival times and/or disease progression often experience a diminished quality of life, potentially impacting the reliability of the LFK index analyses due to varying RR reports across sub-groups. Irinotecan, etoposide, and 5-FU/LV exhibited safety profiles commensurate with the observed toxicities in this trial overall. Notably, the FOLFIRI combination demonstrated minimal and acceptable incidences of haematological toxicity, rendering it well tolerated. Gastrointestinal toxicity, a known side effect of FOLFIRI therapy, may necessitate hospitalisation and immediate medical attention [45]. However, overall toxicity reports may be lower compared to other studies, possibly attributable to the inclusion of studies employing weekly irinotecan (80 mg/m^2^) alongside an AIO-based regimen of 24 h high-dose 5-FU (2300 mg/m^2^) preceded by 2 h LV 500 mg/m^2^, with 44% of patients experiencing grade III/IV [45].

In small cell lung cancer (SCLC) studies, irinotecan-based combinations demonstrated significantly longer overall survival than topotecan-based combinations. These trials primarily enrolled patients with relapsed SCLC, predominantly with favourable Eastern Cooperative Oncology Group (ECOG) statuses. Predominantly phase II cases with substantial objective response rates underscore the efficacy of salvage chemotherapy with irinotecan. Notably, other parameters, including disease control rate, were comparable, suggesting both combinations as viable options. The heightened AEs on haematological parameters observed with topotecan could be linked to the prevalent topotecan dose of 1.5 mg/m^2^ in most patients, representing an overdose, particularly in relapsed SCLC patients, thereby depleting bone marrow reserves. This study revealed significantly higher levels of anaemia, neutropenia, and leucopenia than irinotecan-based combinations. Notably, some authors have explored trials employing lower dose ranges of 1.0–1.25 mg/m^2^ [59]. Irinotecan-based combinations were predominantly paired with cisplatin, etoposide, carboplatin, and bevacizumab. However, cisplatin-based combinations emerged as the most frequently observed and demonstrated superior efficacy compared to topotecan-based combinations. Additionally, the overall toxicity profile favoured irinotecan-based combinations. These findings align with previous meta-analyses suggesting that topotecan-based therapy does not outperform other agents in relapsed SCLC. However, weak evidence indicates its inferiority to platinum-based combinations in sensitive diseases [90]. Previous research has shown that the irinotecan and cisplatin combination was unsuccessful in phase III trials conducted in SCLC patients from the USA, Canada, the Republic of Korea, and Australia [66,78,91]. The study’s limitations included missing data in some studies, preventing a comprehensive analysis. For example, the impact of pharmacogenetics on treatment outcomes and specific details regarding first-line treatments were not available in cases where the studies only reported on second-line treatments. Advances in targeted drug delivery systems—particularly those employing nanotechnology and sophisticated combinational formulations—offer promising solutions to longstanding challenges with CPT bioavailability and delivery. Despite these obstacles, CPT derivatives show immense promise as cancer therapies due to their strong cytotoxic effects on cancer cells. Extensive efforts have been devoted to optimising CPT derivatives through structural modifications to gain mechanistic insights, developing innovative targeted delivery systems to enhance CPT’s pharmacological properties, and overcoming existing limitations [9,10]. This meta-analysis offers critical insights into irinotecan-based chemotherapy, highlighting considerable efficacy in NSCLC with an objective response rate of 31.8% across 23 studies. Irinotecan combined with cisplatin consistently demonstrated superior response rates compared to alternative drug pairings, supporting its preferential use for NSCLC. Despite significant heterogeneity and asymmetry across studies, the results affirm irinotecan’s efficacy in improving progression-free survival, particularly with combinations involving cisplatin and carboplatin. However, elevated toxicity, especially neutropenia, necessitates cautious clinical application. For COLRC and SCLC, irinotecan with bevacizumab and fluorouracil showed optimal survival benefits, but toxicity risks remain a significant challenge. Likewise, the combination therapy of irinotecan with folinic acid and fluorouracil proved most effective for treating O/GC. This review explains the need for tailored therapeutic strategies to balance efficacy and safety across cancer types.

## 5. Conclusions

In conclusion, trials involving camptothecin derivative-based combinations are ongoing and remain the standard treatment regime for cancer therapy. The future of cancer treatment is poised for significant advancements through several emerging trends. Genomic, proteomic, and pharmacogenomic innovations will enable personalised treatments based on individual tumours’ genetic and molecular profiles. Immunotherapy and the application of artificial intelligence and machine learning in analysing large datasets from clinical trials and genetic studies will enhance treatment precision. Liquid biopsies will improve early detection and real-time monitoring of cancer progression. Clustered, regularly interspaced short palindromic repeats and other gene-editing technologies hold promise for correcting cancer-driving mutations. Combination therapies, integrating modalities like chemotherapy, immunotherapy, and radiation, are expected to improve treatment efficacy. Additionally, advancements in nanotechnology, telemedicine, stem cell research, preventive oncology, and lifestyle interventions will contribute to more effective and comprehensive cancer care.

## Figures and Tables

**Table 1:** Irinotecan-based combinations of chemotherapeutic effects on NSCLC patients.

Combinational Therapy	No. of Studies	Patients Investigated	Average RR%	Average DCR%	Median Age	OS	PFS
IRI + CIS	12	693	34.1	66.8	61	10.5	5.5
IRI + CAR	3	137	25.6	72.3	65	10.6	5.5
IRI + DOC	3	110	25.5	61.8	61	10.8	3.5
IRI + PAC	2	69	30.4	66.7	62	9.2	4.4
IRI + GEM	1	39	12.8	71.8	63	8	3.5
IRI + IFO	1	44	29.5	88.6	65	12.5	5.3

Abbreviations: NSCLC, non-small cell lung cancer; IRI, irinotecan; CIS, cisplastin; CAR, carboplatin; DOC, docetaxel; PAC, paclitaxel; GEM, gemcitabine; IFO, ifosfamide; OS, overall survival rate; PFS, progression-free survival rate.

**Table 2:** Non-haematologic and haematologic responses to irinotecan-based combinations on NSCLC patients.

	Toxicity (%)					
Combinational Therapy	Nausea	Diarrhoea	Anaemia	Neutropenia	Leucopenia	Thrombo
IRI + CIS	19.6	19.6	10.5	37.9	17.2	9.7
IRI + CAR	10.1	7.9	27.3	63.3	35.1	33.1
IRI + DOC	0.0	16.9	3.8	26.4	N/A	0.0
IRI + PAC	1.3	7.6	7.6	20.3	N/A	1.8
IRI + GEM	17.9	N/A	5.1	25.6	12.8	2.6
IRI + IFO	0.0	6.8	4.5	38.6	27.3	0.0

Abbreviations: NSCLC, non-small cell lung cancer; IRI, irinotecan; CIS, cisplastin; CAR, carboplatin; DOC, docetaxel; PAC, paclitaxel; GEM, gemcitabine; IFO, ifosfamide; thrombo, thrombocytopenia.

**Table 3:** Irinotecan-based combinations’ chemotherapeutic effects on COLRC patients.

Combinational Therapy	No. of Studies	Patients Investigated	Average RR%	Average DCR%	Median Age	OS	PFS
FOLFIRI	4	686	44.5	74.5	61	18.7	7.5
CAPIRI	3	189	37.0	73.0	74	14	7.5
BEVFOLFIRI	2	441	57.6	88.0	61	28.6	10.9

Abbreviations: COLRC, colorectal cancer; FOLFIRI, folinic acid + fluorouracil + irinotecan; CAPIRI, capecitabine + irinotecan; BEVFOLFIRI, bevacizumab + folinic acid + fluorouracil + irinotecan; DCR, disease control rate; OS, overall survival rate; PFS, progression-free survival rate.

**Table 4:** Non-haematologic and haematologic responses to irinotecan-based combinations on COLRC patients.

	Toxicity (%)					
	Nausea	Diarrhoea	Anaemia	Neutropenia	Leukopaenia	Vomiting
FOLFIRI	3.8	13.7	1.3	20.4	5.0	5.0
CAPIRI	N/A	28.7	6.4	16.4	N/A	6.4
BEVFOLFIRI	4.7	9.8	45.1	31.4	11.3	3.8
	Common treatment plans across studies					
FOLFIRI	FA 500 mg/m^2^ as a 2 h infusion and FU 2.6 g/m^2^ by intravenous 24 h infusion, irinotecan 80 mg/m^2^					
CAPIRI	Irinotecan 80 mg/m^2^ i.v. days 1 and 8 and capecitabine 1000 mg/m^2^ orally b.i.d. days 1–14; q21d					
BEVFOLFIRI	Bevacizumab (5 mg/kg) followed by FOLFIRI (irinotecan 150–180 mg/m^2^; leucovorin 200 mg/m^2^; i.v. bolus of fluorouracil 400 mg/m^2^, continuous infusion of fluorouracil 2400 mg/m^2^)					

Abbreviations: COLRC, colorectal cancer; FOLFIRI, folinic acid + fluorouracil + irinotecan; CAPIRI, capecitabine + irinotecan; BEVFOLFIRI, bevacizumab + folinic acid + fluorouracil + irinotecan.

**Table 5:** Comparison of camptothecin derivative-based combinations on SCLC patients.

Camptothecin Derivatives with/Without Other Drugs Class Against SCLC												
Drug of Interest	No of Studies		Patients Investigated		Average RR%, X^2^, *p*		Average DCR%		Median Age (Yrs)	PFS (Months)		OS (Months)
Topotecan combination	14		1251		26.8	177.2, *p* < 0.00	64.5		63	3.8		7.0
Irinotecan combination	16		1453		52.0 *		74.5		64	5.0		10.3
	Responses (%, X^2^, *p*_value_)											
	CR		PR		SD		PD					
Topotecan combination	3.7		25.0	126.8, *p* < 0.00	32.9 *	14.8, *p* < 0.00	25.8 *	87.8, *p* < 0.00				
Irinotecan combination	5.5		50.3 *		24.4		10.6					
	Toxicity (%, X^2^, *p*_value_)											
	Nausea		Diarrhoea		Anaemia		Neutropenia		Leukopaenia	THROMB		
Topotecan combination	1.5	37.7, *p* < 0.00	1.9	66.5, *p* < 0.00	27.2 *	13.6, *p* < 0.00	67.4 *	115.8, *p* < 0.00	45.1	39.4 *	20.9, *p* < 0.00	
Irinotecan combination	10.0 *		14.5 *		20.1		42.8		39.7	24.7		

* *p* < 0.001. Abbreviations: SCLC, small cell lung cancer; *p*, *p* value; X^2^, chi-square; THROMB, thrombocytopenia; RR, response rate; DCR, disease control rate; PFS, progression-free survival rate; CR, control response; PR, partial response; SD, stable disease; PD, progressive disease.

## Data Availability

The datasets generated during and/or analysed during the current study are available from the corresponding author upon reasonable request.

## Supplementary Materials

The following supporting information can be downloaded at https://www.mdpi.com/article/10.3390/cancers16223802/s1, Table S1: Prisma 2020 checklist; File S1: Total number of references used. Reference [92] is cited in the Supplementary Materials.
