# Supplementary material for: Camptothecin and Its Derivatives from Traditional Chinese Medicine in Combination with Anticancer Therapy Regimens: A Systematic Review and Meta-Analysis
Source: Cancers (Basel). 2024 Nov 12;16(22):3802. doi: 10.3390/cancers16223802 (PMC11593076; doi:10.3390/cancers16223802)
Supplement: Supplementary file 1 [file cancers-16-03802-s001.zip › Supplementary File S1.pdf]

References	Total case	RR (%)	Complete	Partial R	Stable Dsz	Progressiv	Total deat
Kim et al. 2019	173	62,4	2	106	24	4	
Noda et al. 2002	75	86,7	2	63	2	3	
Lara et al. 2009	324	60				32	
Shi et al., 2015	30	70	2	19	4	5	
Schmittel et al. 2011	106	53,8	4	53	20	27	
Zatloukal et al. 2010	202	39,1	8	71	77	10	
Allen et al. 2014	87	0	0	0	14	62	
Huber et al. 2006	164	76,8	31	95	9	29	
Inoue et al., 2008	30	13	0	4	10	16	
Jotte et al., 2011	26	15,4	1	3	9	9	
O'Brien et al. 2006	71	7	0	5	31	24	
Park et al. 2008	17	52,9	1	8	3	5	
von Pawel et al. 2014	213	16,9	1	35	95	44	
Baize et al. 2020	81	23,5			8		
Chiappori et al. 2016	15	13,3	0	2	4	9	
Goto et al. 2016	86	26,7	0	23	40	21	
Goto et al. 2016	83	84,3	9	61	11	0	
Evans et al. 2015	79	10,1	0	8	50	18	
Edelman et al. 2022	94	20,2					
Edelman et al. 2022	190	18,9					
Borghaei et al. 2022	148	21					
Eckardt et al. 2007	151	33	0	33	35	65	
Kang et al. 2021	76	21,1	0	16	38	20	
Schuette et al. 2005	35	17	2	4	7	22	
Ohyanagi et al. 2008	30	36,7	0	11	10	7	
Chen et al. 2009	38	50	5	14	14	5	
Xenidis et al. 2011	26	15,4	0	4	2		
Trafalis et al. 2016	28	25	1	6	18		
Kondo et al. 2018	30	41,3	1	11	8	9	
Kinoshita et al. 2006	61	84	17	34	9	1	
Hirose et al. 2003	22	68,2	0	15	4	3	

ED by PD	Median ag	Plt-Based	NonPlt-Ba:	Early tox	Tox death	Prior Thera	Further the	Radiothera	Surgical re
	66								
	63								
	60				03/106			37/106	
	60								
01/87									
	61	104/164	60/164					85/164	17/164
	64								
	60					46/71	42/70	38/71	18/71
	68								
	61					106/213			
	65							47/81	
	64								
	64							38/90	
	64							42/90	
	62								
	62								
	63,6	30/35	05/35						
	65							13/30	
	65								
	64								
	63,5							06/28	
	67								
	68								
	67								

2nd ther	3rd ther	Haem tox	GI tox (%)	Nausea(%)	Diarrhoea	Stomatitis	Anaemia	Neutroper	Leucopeni
				07/167	17/167		45/167	104/167	
				10/75	12/75		20/75	49/75	20/75
				59/317	60/317		18/317	107/317	
				04/30	05/30		09/30	16/30	
08/106	02/106			09/106	15/106		18/106	04/106	
				20/202	35/202	0/202	14/202	77/202	
								22/87	21/87
					01/30	0/30	09/30	26/30	
					01/23		07/23	18/23	
33/70	10/70								
				01/17	0/17	0/17	02/17	11/17	07/17
							60/197	106/197	43/197
				0/81	02/81		17/81	20/81	
				0/14	01/14		01/14	04/14	04/14
				02/90	0/90		25/90	77/90	46/90
				01/90	07/90		76/90	75/90	72/90
				0/88	0/88		23/88	69/88	57/88
				04/151	04/151		46/151	130/151	113/151
				01/81	02/81		20/81	59/81	18/81
				03/35	05/35	0/35	03/35	02/35	05/35
				0/30	03/30		01/30	13/30	02/30
				05/40	08/40		06/40	22/40	
					01/31		01/31	0/31	
				0/28	0/28		0/28	02/28	03/28
				0/30	03/30		04/30	11/30	
				05/61	08/61		24/61	45/61	20/61
				0/24	05/24		16/24	15/24	14/24

Anorexia	Thromboc	Fatigue	Vomitting	I organ	2 organs	> 2 organs	3 organs	4 organs	OS
			5/167						10,9
									12,8
			33/317						9,9
			04/30						18,1
									10
			22/202	14/202	59/202		77/202	52/202	10,2
	17/87								
				69/164	50/164		33/164	12/164	23.4 wks
0/30		02/30	1/30						
02/17		08/17	0/17	05/17					3,4
		24/197							7,8
01/81	29/81		02/81						7,4
0/14	04/14	01/14	0/14						5,3
04/90	25/90	01/90	0/90						12,5
04/90	37/90	02/90	0/90						18,2
01/88	40/88	63/88	0/88						6,8
									3,4
									3
									8,6
04/151	65/151	12/151							8,2
03/81	36/81	03/81	01/81						8,2
	04/35		0/35						5,8
	01/30								14,4
	09/40								10
	0/31	07/31	0/31	01/31	08/31	22/31			3,2
	0/28	01/28	0/28						6
02/30	01/30		0/30						10,4
	25/61								15
	14/24								6,5

PFS (mont	Medicatio	Sensitive	Refractory	ECOG 0	ECOG 1	ECOG 2	Site	0 month-o	3 month
6,5	Irinotecan+Cisplastin			16/173	132/173	25/173	SCLC		
6,9	Irinotecan+Cisplastin			10/77	61/77	06/77	SCLC		
5,8	Irinotecan+Cisplastin						SCLC		
6	Irinotecan+Cisplastin			03/30	24/30	03/30	SCLC		
6	Irinotecan+Cisplastin						SCLC		
5,4	Irinotecan+Cisplastin			47/202	153/202	02/202	SCLC	202	173
	Topotecan w/wt plantinium						SCLC		
	Topotecan						SCLC		
	Topotecan	04/19	0/11	17/30	09/30	04/30	SCLC		
	Topotecan			10/26	14/26	02/26	SCLC		
	Topotecan			08/71	44/71	19/71	SCLC		
1,7	Topotecan			01/17	13/17	03/17	SCLC		
3,5	Topotecan			72/213	137/213	04/213	SCLC		
4,7	Topotecan			26/81	48/81	07/81	SCLC		
3	Topotecan	07/15	08/15			03/15	SCLC		
3,6	Topotecan			40/90	47/90	03/90	SCLC	90	
5,7	Cisplastin+Etoposide+Irinotecan			52/90	36/90	02/90	SCLC	90	
3	Topotecan						SCLC		
7,4	Topotecan						SCLC		
7	Irinotecan						SCLC		
4,3	Topotecan						SCLC		
3,4	Topotecan			35/151	98/151	18/151	SCLC		
3,8	Topotecan						SCLC	76	
3,4	Irinotecan	02/20	04/15	09/35	21/35	05/35	SLCL		
3	Irinotecan	09/20	02/10	11/30	16/30	03/30	SLCL		
N/A	Irinotecan+Carboplatin						SLCL		
1,9	Irinotecan+liposomal Doxorubici			05/31	21/31	05/31	SLCL		
3	Irinotecan+Bevacizumab						SLCL		
4,1	Irinotecan	11/18	01/12	15/30	15/30		SLCL		
6,1	Irinotecan+Carboplatin			19/61	38/61	04/61	SCLC		
3,9	Irinotecan+Carboplatin			02/24	13/24	09/24	SCLC		

6 month	9 month	12 month	15 month	18 month	21 month	24 month	27 month	30 month	33 month
152	107	72	54	43					
74		49		27		10			
85		68		45		23			
		20				6			

36 month	Phase	Therapy st	0mth-pfs	3mth	6mth	9mth	12mth	15 mth	18 mth
	II								
	II								
	II								
	III								
	III								
	III								
	II								
	II								
	II								
	II								
	II								
	II								

21 mth	24 mth	27 mth	30 mth	33 mth	36 mth
